# *Cantharellus violaceovinosus*, a new species from tropical *Quercus* forests in eastern Mexico

**DOI:** 10.3897/mycokeys.32.22838

**Published:** 2018-03-20

**Authors:** Mariana Herrera, Victor M. Bandala, Leticia Montoya

**Affiliations:** 1 Red Biodiversidad y Sistemática, Instituto de Ecología A.C., P.O. Box 63, Xalapa, Veracruz, 91000, México

**Keywords:** Cantharellales, ectomycorrhizal fungi, Neotropical fungi, oak forest, wild edible mushrooms

## Abstract

During explorations of tropical oak forests in central Veracruz (eastern Mexico), the authors discovered a *Cantharellus* species that produces basidiomes with strikingly violet pileus and a hymenium with yellow, raised gill-like folds. It is harvested locally and valued as a prized edible wild mushroom. Systematic multiyear sampling of basidiomes allowed the recording of the morphological variation exhibited by fresh fruit bodies in different growth stages, which supports the recognition of this *Cantharellus* species from others in the genus. Two molecular phylogenetic analyses based on a set of sequences of species of all major clades in *Cantharellus*, one including sequences of the transcription elongation factor 1-alpha (tef-1α) and a combined tef-1α and nLSU region (the large subunit of the ribosome), confirm the isolated position of the new species in a clade close to *C. lewisii* from USA, in the subgenus Cantharellus. Detailed macroscopic and microscopic descriptions, accompanied by illustrations and a taxonomic discussion are presented.

## Introduction

The diversity of species in *Cantharellus* Adans.: Fr., in combination with their ectomycorrhizal nature (mycobionts of several plant lineages), as well as their highly prized value as edible wild mushrooms, have attracted the attention of specialists from different fields worldwide (Smith and Morse 1947, Trappe 1962, Chandra 1989, Molina et al. 1992, Homola 1993, Thoen 1993, Watling 1997, Danell 1999, Pilz et al. 2002, Lee et al. 2003,Yun and Hall 2004, Boa 2005, Agerer 2006, Arora and Dunham 2008, Kumari et al. 2011, Wilson et al. 2012).

*Cantharellus* encompasses fungi with long-lived, gymnocarpic, fleshy, variedly coloured, trumpet-shaped basidiomes with nearly smooth, veined, gill-like folded to distinctly lamellate hymenophore, pileipellis poorly differentiated, cystidia lacking, smooth and thin-walled spores, with or without clamps (Wilson et al. 2012, Buyck et al. 2014). In many cases, basidiomes of members of closely related species or inclusive, unrelated look-alike species are difficult to identify in a strict sense, especially if there is not an accurate record of the variation of morpho-anatomical characters and colours in fresh condition. Few microscopic features in the genus had been considered discriminative, especially clamps (presence or absence), wall thickness of the terminal elements of the pileipellis hyphae and the basidiospore features (size and form).

The two former features are considered amongst the most taxonomically informative at subgeneric level and the latter used to distinguish species (Eyssartier and Buyck 2001, Buyck et al. 2014). Additionally, it has been hypothesized that there are cryptic species still undefined taxonomically, even amongst the best known *Cantharellus* species, especially from tropical regions but also from temperate regions (Smith and Morse 1947, Corner 1966, Heinemann 1958, 1966, Smith 1968, Petersen and Ryvarden 1971, Petersen 1976, Bigelow 1978, Petersen and Mueller 1992, Buyck and Hofstetter 2011, Buyck et al. 2012, Eyssartier et al. 2003, De Kesel et al. 2011, Tian et al. 2012, Wartchow et al. 2012, Buyck and Randrianjohany 2013, Foltz et al. 2013, Buyck et al. 2014, 2016a, c, Leacock et al. 2016).

Taxonomic research on *Cantharellus* has increased substantially in the last decade, especially by combining DNA and morphological information to support the definition of early recognised species and others recently discovered (Buyck and Hofstetter 2011, Buyck et al. 2011, Tibuhwa et al. 2012, Wilson et al. 2012, Buyck et al. 2012, 2013, 2014, 2015, 2016a, 2016b, Foltz et al. 2013, Shao et al. 2014, Shao et al. 2016, Leacock et al. 2016).

The earliest description of *Cantharellus* in Mexico dates from Fries (1855), who proposed *C. mexicanus* based on a specimen with “… *pileo carnoso turbinato-infundibuliformi glabro griseofusco*… *lamellis augustissimis longe decurrentibus*”. It was collected by F.M. Liebmann at El Mirador, Veracruz and, years later, considered by Corner (1966) as “… *incert. sed.* (? *Gomphus*)…” no longer recorded in the literature. From the same region (Orizaba, relatively near to the current study site), *Craterellus confluens* Berk. & M.A. Curtis was described by Berkeley (1867). This species is characterised by its yellow basidiomes, it is closely related to *Cantharellus lateritius* (Berk.) Singer, with which it has been confused or even with other yellow chanterelles, such as *C. cibarius* Fr. and *Craterellus odoratus* (Schwein.) Fr. (Heim 1954, Corner 1966, Petersen 1979, Guzmán and Sampieri 1984, Buyck and Hofstetter 2011). After such descriptions of new *Cantharellus* species from Mexico, only some additional records of about ten species described from other latitudes have been mentioned to occur in different forest ecosystems in the country, including *Quercus* forests (Guzmán and Sampieri 1984, Guzmán 1985, Guevara et al. 2004, Perez-Moreno et al. 2008, Garibay-Orijel et al. 2009). The identity of these records, however, has not been confirmed with molecular evidence. Recently, *C. coccolobae* Buyck, Moreau & Courtecuisse was described from the Caribbean (Guadeloupe), including two collections from Yucatán, Mexico (Buyck et al. 2016c).

During the authors’ long term explorations in tropical oak forests in central Veracruz, a *Cantharellus* species was found with a striking habit, distinctive when compared to the previous records from Mexico. In fact, this fungus is unique because the fresh basidiomes in different growth stages possess a strikingly violet pileus and yellow, raised gill-like folded hymenophore, in combination with ellipsoid basidiopores and terminal elements of pileipellis slightly thick-walled. The macro- and micromorphological features depicted in this fungus, as well as its distinct position in two phylogenetic analyses, one of tef-1α and other of a combined tef-1α+nLSU sequences datasets, allowed its recognition as a new species. This *Cantharellus* species is locally considered a prized edible mushroom.

## Materials and methods

### Sampling and morphological study

*Cantharellus* basidiomes were collected during June-October, through six consecutive years (2012–2017) including some collections in 2009 and 2011, in tropical oak forests from Zentla (837–850 m a.s.l.) and Alto Lucero (400–500 m a.s.l.) counties in central Veracruz (eastern Mexico). In these oak forests, *Quercus oleoides* is dominant and even forms pure stands. In the Zentla locality, however, *Q. glaucescens* and *Q. sapotifolia* are also present and, at times, also form monodominant small patches. Descriptions of morpho-anatomical features were achieved based on fresh samples and following Largent (1973). The colour notations indicated in the descriptions follow Kornerup and Wanscher (1978) and Munsell colour chart (1994). Basidiomes were dried in a hot air dehydrator (45 °C). Microscopic features were observed and measured after tissues were rehydrated in 3 % potassium hydroxide (KOH) and stained with 1 % Congo red or analysed in Melzer´s solution. At least thirty-five basidiospores per collection were measured in length and width. Mean ranges denoted as *X–m* and the length/width ratio (Q–) of basidiospores, in side view, are given as an interval of mean values per collection (n=15 collections). The form of the basidiospores was interpreted after calculating the Q values, following Bas (1969). Line drawings were made with the aid of a drawing tube. Collections are part of XAL Herbarium (Thiers B. [continuously updated] Index Herbariorum: a global directory of public herbaria and associate staff. New York Botanical Garden`s Virtual Herbarium. http://sweetgum.nybg.org/science/ih/).

### DNA extraction, PCR and sequencing

DNA was isolated from fresh material using DNAeasy Plant Mini Kit (QIAGEN, Hilden, Germany) following the manufacturer´s recommendations. The transcription elongation factor 1-alpha (tef-1α) was amplified using the primers tef1F and tef1R (Morehouse et al. 2003) and the large subunit of ribosome (nLSU) using the primers LR0R and LR7 (Vilgalys and Hesler 1990). PCR conditions were performed with an initial denaturation at 94 °C for 3 min; 35 cycles of 1 min 94 °C, 1 min at 55 °C and 2 min at 72 °C; and final elongation at 72 °C for 7 min. Amplified PCR products were purified with the DNA Clean & Concentrator Kit (Zymo Research, USA) following the manufacturer’s instructions. Cycle sequencing reactions were made using BigDye Terminator 3.1 Cycle Sequencing kit (Applied Biosystems, USA); reactions were purified with ZR DNA Sequencing Clean-up Kit (Zymo Research, USA) and run in a sequencer, ABIPrism 310 Genetic Analyzer (Applied Biosystems). Sequences obtained were assembled and edited in BioEdit (Hall 1999) and deposited at GenBank database (Benson et al. 2017) (Table 1).

**Table 1.** *Cantharellus* species included in this study: samples, location and accession number for tef-1α and nLSU sequences.

Taxon	Voucher Specimen	Location	GenBank	
			*tef-1*α	nLSU
*C. addaiensis*	BB 98.033	Tanzania	JX192992	KF294667
*C. afrocibarius*	BB 96.236	Zambia	JX192993	KF294668
*C. albidolutescens*	BB 08.057	Madagascar	KF294752	KF294645
*C. albidolutescens*	BB 08.080	Madagascar	JX192982	–
*C. ambohitantelyensis*	BB 08.336	Madagascar	JX192989	–
*C. amethysteus*	BB 07.284	Slovakia	GQ914953	KF294639
*C. amethysteus*	BB 07.309	Slovakia	GQ914954	KF294642
*C. appalachiensis*	BB 07.123	USA	GQ914979	KF294565
*C. cascadensis*	BB 13.251	USA	KX857044	–
*C. chicagoensis*	JJ/MO-CANT1	USA	KX857025	–
*C. cibarius*	BB 07.300	Slovakia	GQ914950	KF294641
*C. cibarius*	GE 07.025	France	GQ914949	–
*C. cinnabarinus*	BB 07.053	USA	GQ914984	KF294630
*C. cinnabarinus*	BB 07.001	USA	GQ914985	KF294624
*C. congolensis*	BB 98.039	Tanzania	JX193015	KF294609
*C. congolensis*	BB 98.058	Tanzania	JX192996	KF294673
*C. corallinus*	JJ/MO-CANT2	USA	KX857031	–
*C. corallinus*	JJ/MO-CANT5	USA	KX857034	–
*C. deceptivus*	JJ/NC-CANT5	USA	KX857029	–
*C. decolorans*	BB 08.278	Madagascar	GQ914968	–
*C. decolorans*	BB 08.243	Madagascar	JX192987	–
*C. densifolius*	BB 98.013	Tanzania	JX193014	KF294616
*C. ferruginascens*	BB 07.283	Slovakia	GQ914952	KF294638
*C. fistulosus*	DT 43	Tanzania	JX192997	KF294674
*C. flavolateritius*	VH 1076	USA	KX857027	–
*C. flavolteritius*	VH1078	USA	KX857029	–
*C. gracilis*	BB 98.234	Tanzania	JX192970	–
*C. humidicolus*	BB 98.036	Tanzania	JX193005	KF294666
*C. ibityensis*	BB 08.203	Madagascar	JX192985	KF294651
*C. isabellinus var. parvisporus*	BB 98.020	Tanzania	JX192972	KF294614
*C. iuventateviridis*	SH13/7/2012	USA	KX857063	–
*C. iuventateviridis*	SH14/7/2012	USA	KX857064	–
*C. lateritius*	BB 07.025	USA	GQ914957	KF294628
*C. lateritius*	BB 07.058	USA	GQ914959	KF294633
*C. lewisii*	BB 02.197	USA	GQ914961	KF294623
*C. lewisii*	BB 07.003	USA	GQ914962	–
*C. lilacinopruinatus*	BB 07.221	Slovakia	GQ914951	KF294637
*C. minor*	BB 07.002	USA	JX192978	KF294625
*C. minor*	BB 07.057	USA	JX192979	KF294632
*C. pallens*	BB 09.441	Italy	KX857013	–
*C. pallens*	BB 12.082	Italy	KX857035	–
*C. paucifurcatus*	BB 08.320	Madagascar	KF294655	JK192988
*C. persicinus*	MH 15.001	USA	KX857080	–
*C. phasmasis*	CO57	USA	JX030417	–
*C. phasmasis*	CO74	USA	JX030418	–
*C. platyphyllus*	BB 98.012	Tanzania	GQ914969	KF294617
C. platyphyllus subsp. bojeriensis	BB 08.160	Madagascar	JX192984	KF294648
*C. pseudominimus*	JV 00.663	Portugal	JX192991	KF294657
*C. quercophilus*	BB 07.097	USA	JX192981	KF294644
*C. sebosus*	BB 08.234	Madagascar	JX192986	KF294652
*C. spectaculus*	C081	USA	JX030414	–
*C. cf subamethysteus*	AV 12.003	Thailand	KX857062	–
*C. subcyanoxanthus*	BB 00.1137	Madagascar	JX192990	–
C. subincarnatus subsp. rubrosalmoneus	BB 06.080	Madagascar	JX192962	KF294601
C. subincarnatus subsp. rubrosalmoneus	BB 06.096	Madagascar	JX192963	KF294602
*C. symoensii*	BB 98.011	Tanzania	GQ914970	KF294618
*C. symoensii*	BB 98.113	Tanzania	JX192974	KF294619
*C. tabernensis*	BB 07.119	USA	GQ914976	KF294634
*C. tabernensis*	BB 07.020	USA	GQ914971	–
*C. tanzanicus*	BB 98.040	Tanzania	JX192977	KF294622
*C. tenuithrix*	BB 14.008	USA	KX857045	–
*C. tenuithrix*	BB 14.009	USA	KX857045	–
*C. tomentosus*	BB 98.038	Tanzania	GQ914965	KF294610
*C. vellutinus*	VH 1583	USA	KX857070	–
*C. vellutinus*	WR WV 07.074	USA	KX857068	–
*C. versicolor*	Tian 160	China	KM893857	–
*C. versicolor*	Yu 24	China	KM893856	–
C. violaceovinosus*	Bandala 4513	Mexico	MF616520	MF616524
C. violaceovinosus*	Corona 648	Mexico	MF616521	MF616525
C. violaceovinosus*	Herrera125	Mexico	MF616522	MF616526
*Craterellus tubaeformis*	BB 07.293	Slovakia	GQ914989	KF294640
*Hydnum repandum*	BB 07.341	Slovakia	JX192980	KF294643

*samples and sequences obtained here

### Phylogenetic analysis

Six tef-1α and nLSU sequences obtained in this study, together with 113 sequences of *Cantharellus* species from all major clades across the genus (after Buyck et al. 2014) and with the highest similarity scores from the results of BLAST (Altschul et al. 1997) were downloaded from GenBank (http://www.ncbi.nlm.nih.gov/) and used to construct two datasets. One dataset consisted of tef-1α and other combined tef-1α+nLSU sequences. *Craterellus tubaeformis* and *Hydnum repandum* were included as outgroup taxa (Table 1 and alignment in TreeBASE S21920). Both datasets were assembled in the data editor PhyDE v.0.995 programme (Müller et al. 2010). They were aligned using Muscle (Edgar 2004) with inconsistencies corrected manually. A phylogeny of each dataset was constructed under maximum likelihood (ML) and Bayesian Inference (BI) methods. The best evolutionary model for both datasets was calculated with Mega 6.06 (Tamura et al. 2013). ML analyses were also performed using Mega 6.06 with 500 replicates of bootstrap. BI analyses were implemented with MrBayes on XSEDE (3.2.6) on CIPRES portal (Miller et al. 2010) with settings as described in Montoya et al. (2014). The phylogenies from ML and BI analyses were displayed using Mega 6.06 and FigTree v 1.3.1 (Rambaut 2009), respectively.

## Results

**Figure 1.**
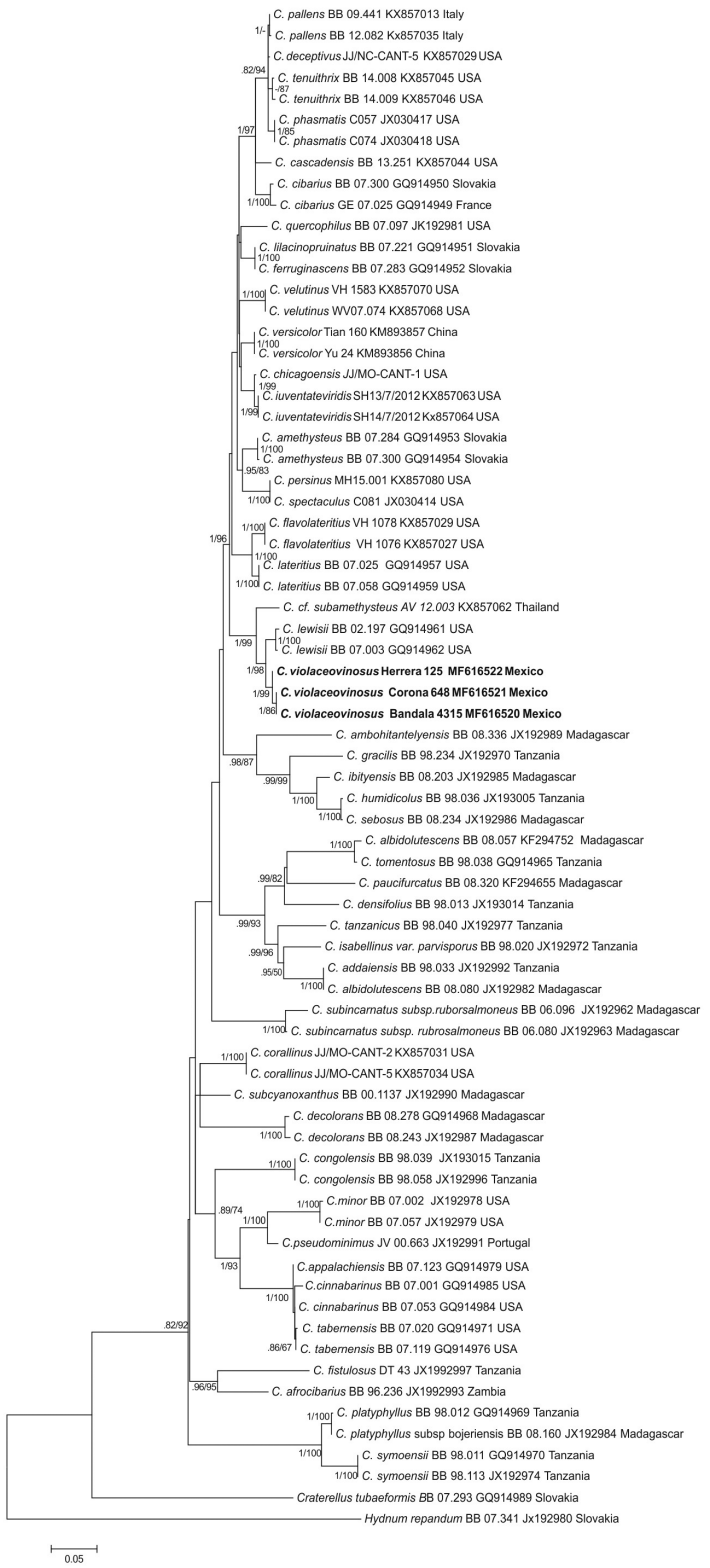
Molecular phylogenetic analysis by maximum likelihood of tef-1α sequences dataset of *Cantharellus* species. Posterior probabilities and Bootstrap values (BPP/BS) are indicated on the tree branches.

**Figure 2.**
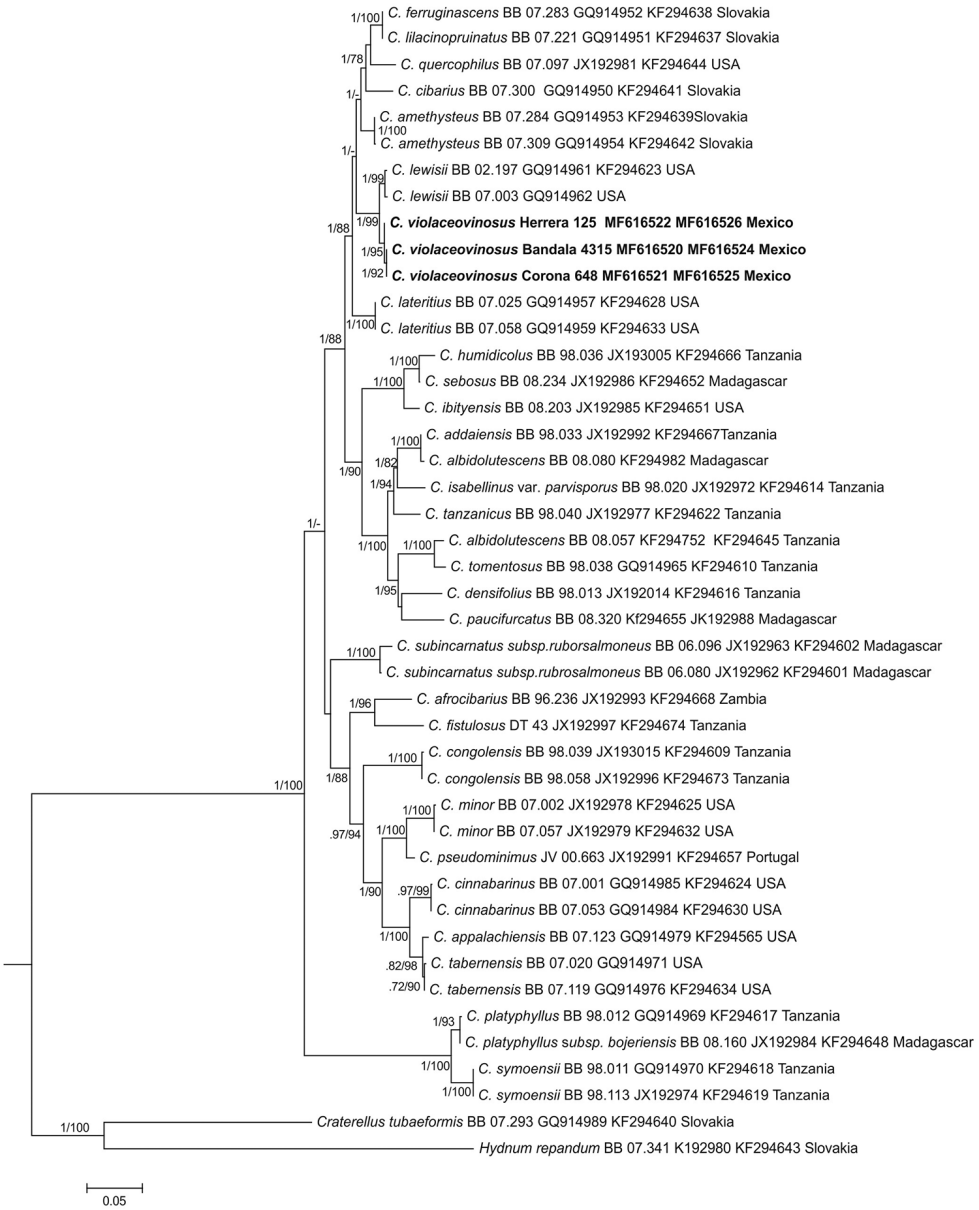
Molecular phylogenetic analysis by maximum likelihood of tef-1α+nLSU sequences dataset of *Cantharellus* species. Posterior probabilities and Bootstrap values (BPP/BS) are indicated on the tree branches.

Sixty fresh collections were obtained of the violet *Cantharellus* species, including basidiomes in different growth stages, most of them detected between August-October, in both localities explored. Six new tef-1α and nLSU sequences from three collections were generated in this study (Table 1). In the inferred molecular phylogenies (from tef-1α and tef-1α+nLSU sequences datasets) (Figs 1–2), the generated sequences from the Mexican specimens, clustered in a terminal clade, strongly supported only bootstrap values ≥70 and posterior probabilities ≥0.90 were considered and indicated (BS/BPP) on the branches of each tree. Both trees were congruent and the sequences of the Mexican *Cantharellus* cluster in a sister clade to *C. lewisii* from USA, in the subgenus Cantharellus (Buyck et al. 2014). Based on the distinctive morphological features and colour variation of the studied *Cantharellus* specimens, as well as the isolated position of the samples in the phylogenies obtained, it was concluded that this should be proposed as a new *Cantharellus* species, which inhabits the tropical *Quercus* forests in eastern Mexico.

### Description of the new species

#### Cantharellus violaceovinosus

Taxon classificationFungiCantharellalesCantharellaceae

M. Herrera, Bandala & Montoya sp. nov.

MycoBank: MB823600

Figs 3 , 4 , 5

##### Holotype.

MEXICO. Veracruz: Municipality of Zentla, around town of Zentla, 850 m a.s.l., gregarious in soil, under *Quercus oleoides* Schltdl. & Cham., 5 July 2012, Corona 648 (XAL).

##### Diagnosis.

Differing from other *Cantharellus* species by: uniformly dark violet, violet-grey to violet-wine or violet-reddish pileus; yellow, gill-like folded hymenophore and ellipsoid basidiospores 7–10 (–11) × (4.5–) 5–6.5 (–7) µm. *X–m* = 7.8–9× 5.1–6.3 µm, Q–= 1.31–1.66, basidia (40–) 45–114 (–125) × (6–) 7–11 (–12) µm, with (1–) 2–5 sterigmata, and terminal elements of the pileipellis 4–6 µm diam, slightly thick-walled.

##### Gene sequences ex-holotype.

MF616521 (tef-1α), MF616525 (nLSU).

##### Etymology.

Referring to the dark violaceous, becoming wine to reddish pileus.

*Pileus* (15–) 25–113 mm diam, convex to broadly-convex with margin incurved when young, expanding to plane or subplane, often shallowly depressed or finally broadly infundibuliform, surface dry, not hygrophanous, dull, smooth, glabrescent, surface at times breaking in faintly tesselate-rimose-like pattern, then appearing appressed fibrillose with age and not forming scales; surface uniformly dark violet (15D4, 15F2–7, 16D3–4, 16D6, 16F4–5) to pale violet with age (15DE5–7) or violet-grey (16D3–4, 16D6), lilac or greyish-lilac (15A3, 15C3–4, 16C2–3), becoming violet-wine or violet-reddish (14E5–8, 14EF4–5), wine (12D4), fading with age and sun exposure, developing pinkish, lilac and reddish tints, especially towards the margin (13A3–4, 13D3–4, 15A4–5), naked parts showing the yellow context (4A2–3); margin incurved or straight, entire or slightly crenate, undulate or irregular, often incised, rarely lobed, not striated. *Hymenophore* with well-defined gill-like folds, up to 3 mm deep, decurrent, subdistant, in some specimens almost straight and inclusive thin, in other materials with faintly sinuous or irregular thicker folds, frequently forking at different levels or only towards the pileus margin, with lower irregular anastomosis amongst the folds, in some specimens the anastomosis occur practically in the whole hymenophore, while in others only at some areas, especially at pileus margin, some specimens (specially towards the stipe) with irregular low veins amongst the folds or the folds become as low and sinuous vein-like; butter-yellow or yellow (2.5Y 8/4,10YR 8/6; 4A3–4). *Stipe* (20–) 25–75 × 5–18 mm, equal and only slightly swollen at base or widening above and tapering gradually downwards, solid, surface glabrous, concolorous with hymenophore, often staining ochraceous or rusty orange colour when handled, occasionally with whitish, small rhizomorphs at base. *Context* whitish to yellow (4A2–3), at times wax-like, odour mild, agreeable, at time fruity somewhat to apricot; taste mild, agreeable.

*Basidiospores* 7–10 (–11) × (4.5–) 5– 6.5 (–7) µm, [*X–m* = 7.8–9 × 5.1–6.3 µm, Q–= 1.31–1.66, (n=13)], ellipsoid, smooth, thin-walled, hyaline, inamyloid, devoid of granular contents or refractive droplets. *Basidia* (40–) 45–114 (–125) × (6–) 7–11(–12) µm, narrowly clavate to subcylindrical, with (1–) 2–5 sterigmata 8–10 µm long, thin-walled, hyaline; subhymenium composed of cylindrical hyphae 4–5 µm diam. *Cystidia* absent. *Pileipellis* a cutis composed of hyphae 4–6 µm diam, intermingled in a compact arrangement, cylindrical, hyaline to yellowish, inamyloid, often some of them with pale brownish contents, these decidedly brown coloured in group; distinctive terminal elements 4–6 µm broad, slightly thick-walled (<1 µm thick), smooth, hyaline, some pale brownish, scattered on the surface. *Pileus trama* composed of cylindrical to inflated hyphae, 3–12 µm diam, slightly thick-walled (<1 µm thick), hyaline, yellowish in mass, some of the hyphal segments completely filled with darker contents. *Hymenophoral trama* composed of hyphae 3–5 µm diam, thin-walled, some with weakly refringent contents. *Clamp connections* present on hyphae in all tissues.

##### Habitat.

Solitary to gregarious, in soil, at tropical oak forest, under *Quercus oleoides*, less frequently also under both *Q. glaucescens* Bonpl. and *Q. sapotifolia* Liebm. June-October, known in the coastal plain of central Veracruz State, east coast of Mexico.

##### Specimen examined.

MEXICO. Veracruz, Zentla Co., Road Puentecilla-La Piña, 837 m a.s.l., 2 Jul 2009, Del Moral 427, Ramos 216; 27 Oct 2009, García 20, García 22; 16 Jun 2011, Bandala 4490; 21 Jul 2012, Herrera 25; 31 Jul 2012, Bandala 4513; 20 Sep 2012, Bandala 4550, Corona 743; 4 Oct 2012, Bandala 4569, 4573; 4 Jul 2013, Gutiérrez 23; 12 Jul 2013, Bandala 4671; 20 Sep 2013, Herrera 67; 15 Sep 2015, Herrera 135. Around town of Zentla, 850 m a.s.l., 5 Jul 2012, Corona 648; 25 Jun 2013, Herrera 60, 61; 15 Sep 2015, Herrera 137, Santillan 16; 1 Oct 2015, Herrera 151; 30 Jun 2016, Herrera 172; 6 Jul 2016, Herrera 184; 12 Jul 2016, Herrera 187; 22 Sep 2016, Herrera 200, 201, 202, 203; 5 Oct 2016, De la Cruz 14,15; 13 Oct 2016, De la Cruz 42; 27 Oct 2016, Herrera 210, 211; 6 Jul 2017, Garay 350; 3 Aug 2017, Garay 364; 31 Aug 17, Garrido 79; 7 Sep 2017, Herrera 214, 215, 216; 15 Sep 17, Montoya 5403; 21 Sep 17, Corona 1420; 5 Oct 17, Mateo 5. Alto Lucero Co., NE Mesa de Venticuatro, 450–500 m a.s.l., 2 Jul 15, Herrera 125, Herrera 126; 17 Sep 2015, Herrera 138; 2 Aug 2016, Herrera 191; 10 Aug 2016, Herrera 192; 20 Sep 2016, Herrera 195, 196, 197, 198; 27 Sep 2016, Herrera 205, 206, 207; 4 Oct 2016, Herrera 208, 209; 22 Aug 17, Herrera 214; 12 Sep 2017, Garay 375; 19 Sep 2017, Garay 392; 2 Oct 17, Mateo 1 (all at XAL).

## Discussion

Distinctive features of this species include the medium to large size basidiomes, with pileus practically homogeneously violet pigmented (only fading with age), smooth, with surface free of scales, at times with disrupted pileus surfaces due to age, hymenophore bearing yellow gill-like folds, ellipsoid, medium-sized basidiospores [7–10 (–11) × (4.5–) 5– 6.5 (–7) µm], medium to large basidia [(40–) 45–114 (–125) × (6–) 7–11(–12) µm] and terminal elements of pileipellis 4–6 µm diam, slightly thick-walled (<1 µm thick). Molecular phylogenetic analyses support that the species is genetically distinct from other *Cantharellus* taxa, in both analyses, *C. violaceovinosus* was nested in an isolated and well-supported clade (95–99/1) (Figs 1–2).

*Cantharellus* species with basidiomata having violet pileus are rare but occur in various regions worldwide (Eyssartier et al. 2009; Buyck et al. 2012). Amongst about 45 species of the genus known from USA, Mexico, Central and South America (Guzmán and Sampieri 1984, Guzmán 1985, Eyssartier et al. 2003, Guevara et al. 2004, Henkel et al. 2006, Wartchow et al. 2012, Wilson et al. 2012, Pinheiro and Wartchow 2013, Wartchow et al. 2013, Nascimento et al. 2014, Buyck et al. 2016c), *C. lewisii* Buyck & V. Hofst., *C. atrolilacinus* Eyssart., Buyck & Halling and the new *C. violaceovinosus* are, up to now, the species known to produce basidiomes with violet tints in the Americas.

*Cantharellus lewisii* grows in floodplain hardwoods, in Water Oak plots next to a *Taxodium* swamp, in beech-magnolia-loblolly pine forests and also under beech-white oak-loblolly pine-magnolia forests in the south of USA (Buyck and Hofstetter 2011). In the inferred phylogeny, it appears as sister of *C. violaceovinosus*, but differs because its pileus is pale yellow, dull to greyish-yellow or ochre to pale brownish-orange, sometimes reddish-brown near the margin, with a surface covered with dark purplish-lilac appressed fibrils (in young stages, *C. lewisii* is often entirely dark lilac-purple) and with terminal elements of pileipellis conspicuously thick-walled (mostly 1–1.5 μm thick) (Buyck and Hofstetter 2011). According to the original description, *C. lewisii* also differs by its ellipsoid or often somewhat reniform and narrower basidiospores [(7.08–) 7.16–7.62–8.07 (–8.96) × (4.17–) 4.24–4.58–4.93 (–5.21) μm; Q= (1.42–) 1.45–1.57–1.70 (–1.80)] and by 5–6-spored and shorter basidia (60–75 × 7–8 μm) (Buyck and Hofstetter 2011). Two Texan collections of *C. lewisii* (holotype BB 07.003 and BB 02.197, both at PC) were studied. Based on observations, it was confirmed that this later species differs from the Mexican *C. violaceovinosus*, because of its markedly reniform, narrower basidiospores, then tending to be “more ellipsoid” [BB 07.003, holotype: 7.5–9.5 × (4–) 4.5–5.5 µm, *X–m* = 8.4 × 5 µm, Q–= 1.68; BB 02.197: 7.5–10 (–11) × 4–5.5 µm, *X–m* = 9 × 4.8 µm, Q–= 1.87).

*Cantharellus atrolilacinus* was described from Costa Rica, growing under *Quercus corrugata* Hook.) and *Q.* sp. (Eyssartier et al. 2003). According to the data on this species (R. Halling, www.nybg.org/bsci/res/hall/canlilac.html; Eyssartier et al. 2003), it differs from *C. violaceovinosus* because its pileus colours tend to be darker, even blackish, dark lilac-grey or brown-lilac, with tomentose surface at the disc, with strong radial, adnate fibrils at the margin, and the stipe whitish with lilac tints. Microscopically, *C. atrolilacinus* has basidiospores (7–) 7.5–8–8.5 (–9) × 4.5–5–5.5 (–6) μm, tending to be more ellipsoid (Eyssartier et al. 2003, fig. 1:2) and having wider pileipellis hyphae [(4–) 5–10 (–15) μm] with a very thick wall (“..très nettement épaissies..”).

Although *Cantharellus amethysteus* (Quél.) Sacc. (subg. Cantharellus) from Europe, may appear superficially similar to some forms of *C. violaceovinosus*, the former however, especially has a pileus surface covered with vinous or lilac, small scales. The authors studied two specimens of *C. amethysteus* from France (BB 07.284 and BB 07.309 at PC) displaying elongate basidiospores, 9.5–12 (–12.5) × 5–7 µm (*X–m* = 11–11.2 × 5.9–6.4 µm; Q–= 1.76–1.86), as Eyssartier and Buyck (2000) reported [(9–) 9.5–10.37–11.5 (–12.5) × 6–6.5–7 µm], resulting in being larger and more elongate than in the Mexican species. Also, it is interesting that one sequence of tef-1α of a specimen from Thailand (GB coded KX857062, Table 1) identified as “C. cf. subamethysteus”, appeared close to *C. violaceovinosus* (Fig. 1). *Cantharellus subamethysteus* indeed is phylogenetically related to *C. lewisii* (Buyck et al. 2014) and differs from *C. violaceovinosus* in the shorter basidiomes (pileus 20–65 mm; stipe 42–57 × 5–11 mm), deep and bright yellow pileus surface, covered with squamules even with rather brown to dark brown tinges, shorter basidiospores [7–8 (8.75) × (4.75) 5–6 μm] and wider pileipellis elements (8–15 μm width) (Eyssartier et al. 2009). Additionally, the hymenophore of this species is rugose to faintly veined (as depicted in the picture accompanying the description).

*Cantharellus goossensiae* (Beeli) Heinem., *C. cyanoxanthus* R. Heim ex Heinem., *C. subcyanoxanthus* Buyck, Randrianjohany & Eyssart. and *C. longisporus* Heinem. represent African species with basidiomes displaying violaceous tinges (Buyck et al. 2012) therefore, at some stages their basidiomes could resemble those of *C. violaceovinosus*. However, the three former species have the pileipellis with thin-walled hyphal extremities, thus differing from members of subgenus Cantharellus, including the new species here described. Moreover, the four African taxa have basidiospores distinctly narrowly ellipsoid to elongate (Q>1.70) and often slightly reniform, curved or even somewhat peanut-shaped (Beeli 1928, Heinemann 1958, 1959, Buyck et al. 2012).

*Cantarellus violaceovinosus* was recorded as a common fleshy mushroom, during the multiyear sampling developed in the tropical *Quercus* forests studied. It was found in ectomycorrhizal association with native trees of *Quercus* species. This mushroom was very often recorded in pure stands of *Q. oleoides* and less frequently in *Q. glaucescens* and *Q. sapotifolia* patches. This violet pigmented chanterelle shares the same habit preferences as *C. lateritius*, also found in the study sites. A similar co-ocurrence has been reported between *C. lewisii* (the sister relative of *C. violaceovinosus*) and *C. lateritius* in the State of Texas in the USA (Buyck et al. 2011). Basidiomes of *C. violaceovinosus* and *C. lateritius* are abundant in the local oak forests studied and both are considered choice wild mushrooms although the latter is more highly prized. They are even more appreciated than species of *Amanita* or *Lactarius*, representing an income source for wild mushroom collectors. Benefits from mushrooms harvesting, as well as other ecosystemic services, are motivating some owners to conserve relicts of the tropical *Quercus* forest of the region.

**Figure 3.**
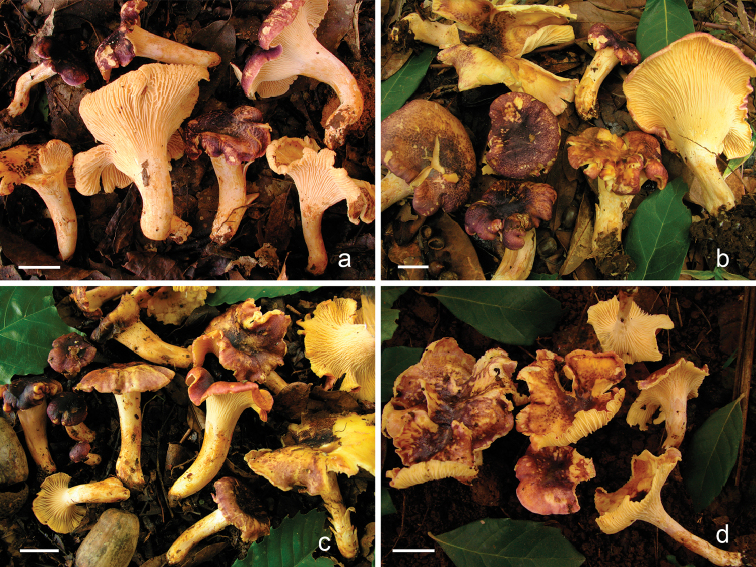
Basidiomes of *Cantharellus violaceovinosus*: **a** Corona 648 (holotype) **b** Bandala 4550 **c** Del Moral 427 **d** Bandala 4490. Scale bars: 20 mm.

**Figure 4.**
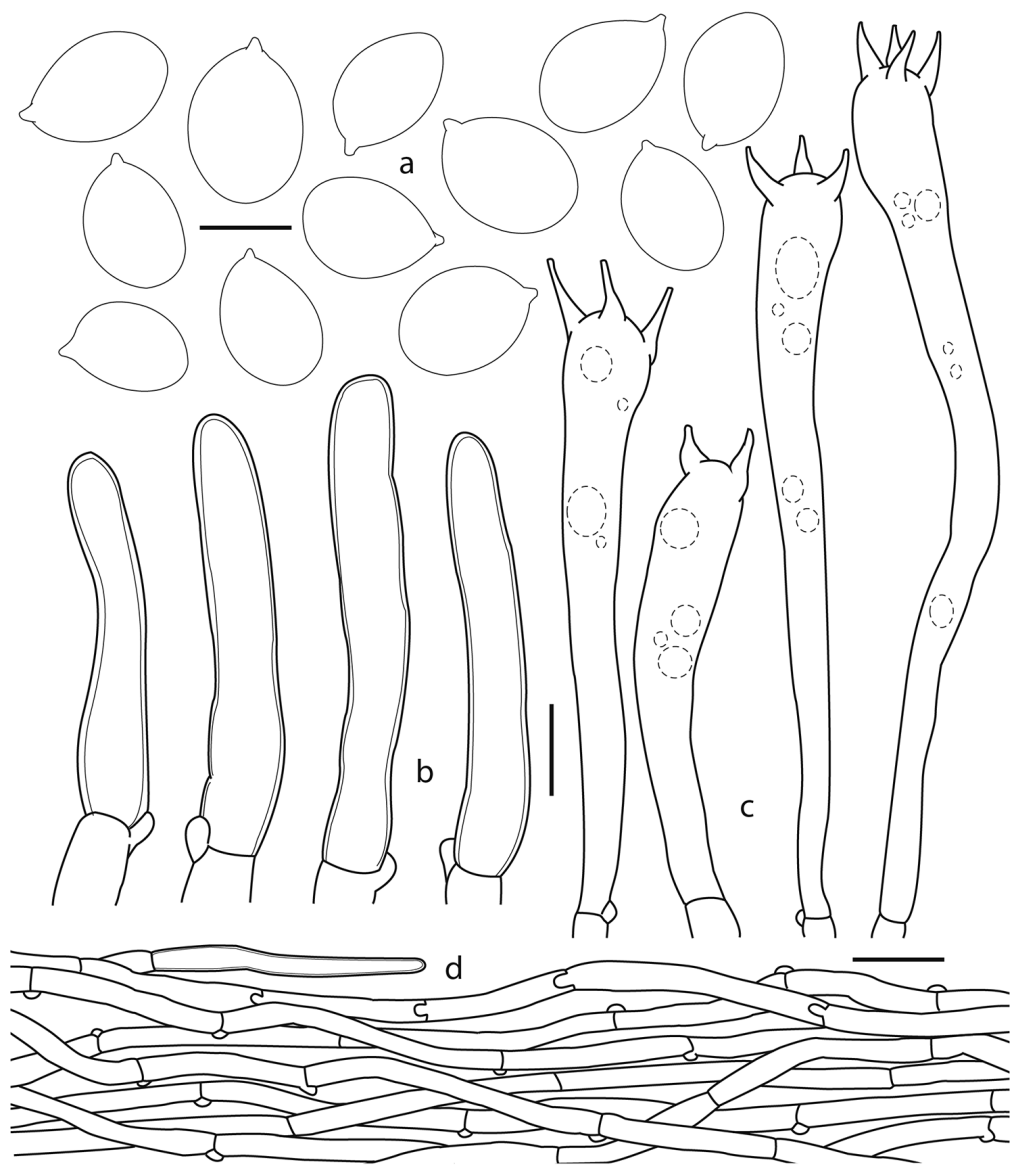
*Cantharellus violaceovinosus* (Corona 648, holotype): **a** basidiospores **b** terminal elements of the pileipellis **c** basidia **d** pileipellis. Scale bars: 5 µm (**a**); 10 µm **(b, c)**; 25 µm **(d**).

**Figure 5.**
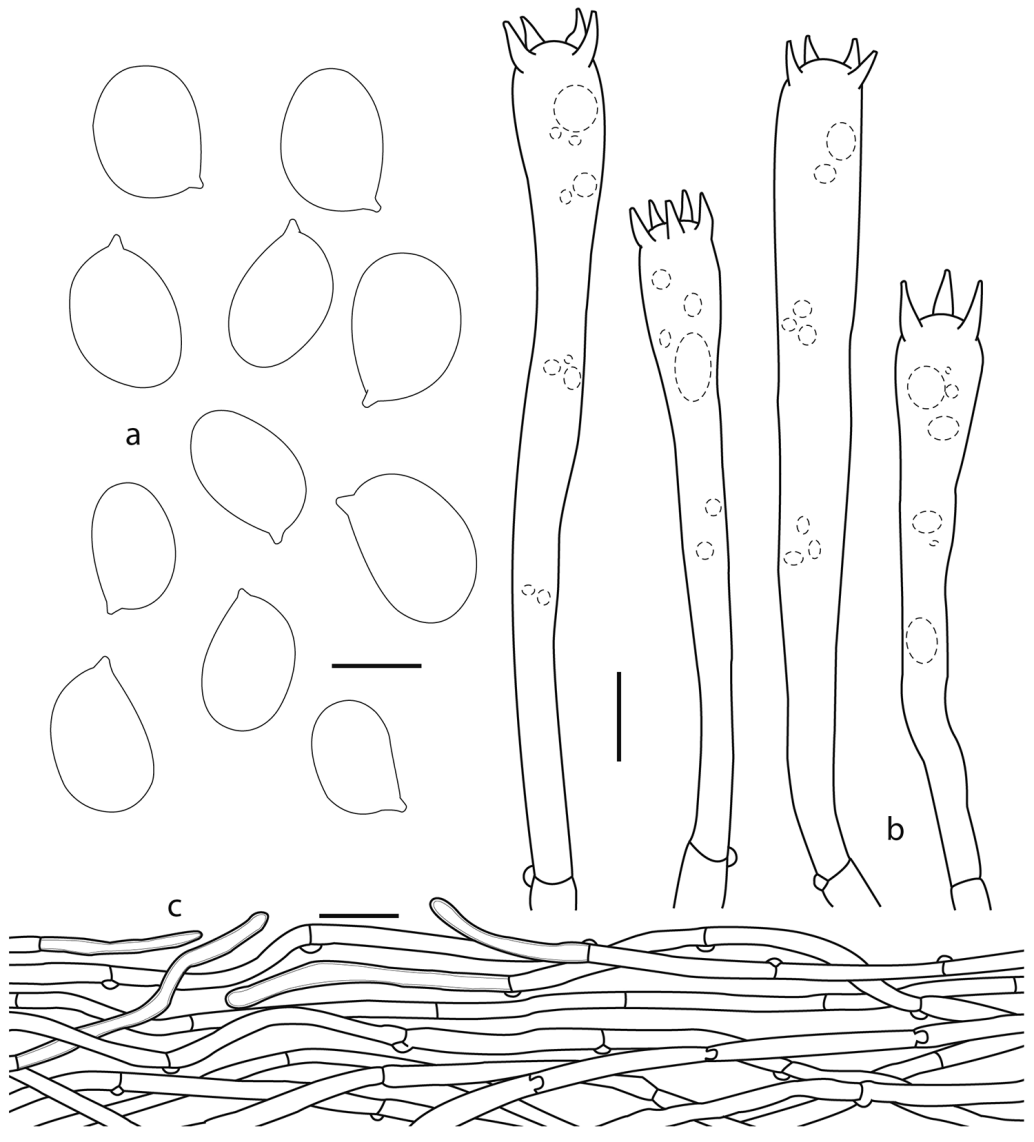
*Cantharellus violaceovinosus*: **a** basidiospores **b** basidia (Gutiérrez 23) **c** pileipellis (Herrera 61). Scale bars: 5 µm (**a**); 10 µm (**b**); 25 µm (**c**).

## Supplementary Material

XML Treatment for Cantharellus violaceovinosus
